# Acute stroke awareness of family physicians: translation of policy to practice

**DOI:** 10.1186/s12961-020-00642-5

**Published:** 2020-10-31

**Authors:** Szilvia Harsanyi, Nandor Balogh, Laszlo Robert Kolozsvari, Laszlo Mezes, Csaba Papp, Judit Zsuga

**Affiliations:** 1 Department of Health Systems Management and Quality Management for Health Care, Faculty of Public Health, University of Debrecen, Nagyerdei krt. 98, Debrecen, 4032 Hungary; 2 University of Debrecen, Doctoral School of Health Sciences, Debrecen, Hungary; 3 Department of Family and Occupational Medicine, Faculty of Public Health, University of Debrecen, Debrecen, Hungary; 4 Institute for Primary Health Care and Health Development of Debrecen, Clinical Centre, University of Debrecen, Debrecen, Hungary

**Keywords:** Acute stroke, Translational research, Word cloud, Primary care

## Abstract

**Background:**

Translating clinical guidelines into routine clinical practice is mandatory to achieve population level improvement of health. Emergence of specific therapy for acute stroke yielded the ‘time is brain’ concept introducing the need for emergency treatment, pointing to the need for increasing stroke awareness of the general population. General practitioners (GPs) manage chronic diseases and could hence catalyse stroke awareness. In our study, the knowledge of general practitioners toward accurate identification of acute stroke candidacy was investigated.

**Methods:**

GPs and residents in training for family medicine participated in a survey on a voluntary basis using supervised self-administration between the 1st of February 2018 and 31st July 2018. Two clinical cases of acute stroke that differed only regarding the patient’s eligibility for intravenous thrombolysis were presented. Participants answered two open-ended questions. Text analysis was performed using NVIVO software.

**Results:**

Of the 127 respondents, 69 (54.3%) were female. The median age was 49 (34–62) years. The median time spent working after graduation was 14.5 (2–22.5) years. Board-certified GPs made up 77.2% of the sample. Qualitative analysis revealed stroke as the most frequent diagnosis for both cases. Territorial localization and possible aetiology were also established. Respondents properly identified eligibility for thrombolysis. Quantitative assessment showed that having the practice closer to the stroke centre increases the likelihood of adequate diagnosis for acute stroke.

**Conclusions:**

Our results show that GPs properly diagnose acute stroke and identify intravenous thrombolysis candidates. Moreover, we found that a vigorous acute stroke triage system facilitates the translation of theory into practice.

## Introduction

Translating scientific evidence into clinical practice is fundamental to attain population level positive health outcomes [1]. Accordingly, several frameworks addressing translational research, including the RE-AIM model [2], the research translation continuum model [3] or the evidence-based public health model [4], emphasize the need for systems level interventions to facilitate the widespread clinical implementation of medical discoveries. A significant step in the ‘bench to bedside’ process concerns the dissemination of interventions, e.g. once clinical practice guidelines disclose evidence-based recommendations, general implementation by the healthcare field is expected [5]. Nonetheless, the gap between policy and routine clinical practice is well acknowledged [6].

The past decade witnessed a paradigm shift in acute stroke management as stroke became acknowledged as an emergency. This was paralleled by the availability of specific treatment by means of intravenous thrombolysis, yielding the ‘time is brain’ concept [7]. Intravenous administration of recombinant tissue plasminogen activator is indicated in ischemic stroke within a limited time window of 4.5 h [8]. This has triggered the organization of triage systems focusing on prehospital emergency medical services (EMS) and hospital management in order to minimize the onset-to-needle, call-to-needle and door-to-needle times [9]. The cornerstone of optimizing acute stroke care concerns raising stroke awareness as one of the main sources of delays comes from the failure to recognize the symptoms and the lack of appropriate action to seek medical help [10]. Evidence shows that help is usually sought by family members and the first contact is often the general practitioner (GP) who will contact the EMS [11]. Additionally, a lack of perceiving stroke as a medical emergency by GPs was identified as one of the reasons why urgent medical attention was not sought at the time of introducing the ‘time is brain’ concept [7]. Furthermore, recently released guidelines still emphasize the benefit of educating the general population [8]. The main points for education were identified as prompt recognition of symptoms, realization of the need for immediate medical attention, the need to use EMS and immediate transport to a stroke centre [7] equipped to perform intravenous thrombolysis in acute stroke patients. According to the guidelines of the American Stroke Association, primary stroke centres have acute stroke teams, written care protocols, coordination with EMS, access to neuroimaging and laboratory services, dedicated stroke units. and access to early rehabilitation [12].

Managing stroke as an emergency was first introduced into the Hungarian guidelines developed by the Hungarian Stroke Association in 2004 [9]. Subsequently, acute stroke triage was formalized in several service areas, including the extended catchment area of the Department of Neurology of the University of Debrecen. Accordingly, a formalized partnership agreement was signed in 2007 with the EMS to transport every thrombolysis candidate within a 90 km radius directly to the acute stroke unit of the Department. Measures to minimize door-to-needle time were instigated as patients were delivered directly to the CT suite, where blood was drawn and the neurologist examined the patient. The programme was augmented by continuous education and training delivered for EMS personnel, GPs, stroke nurses and physicians. There are some good practice examples regarding the implementation of thrombolysis for acute stroke internationally as well [13].

Starting from these considerations, we set out to investigate whether the critical step of translation to dissemination and implementation of evidence-based recommendation in routine clinical care has been undertaken regarding acute stroke management. Given that GPs undertake the management of chronic diseases, many of which are risk diseases for stroke, they could be a key asset for increasing their patients’ awareness regarding acute stroke. However, according to the guidelines, the GP does not assume an active role in acute stroke management, there lies a responsibility for educating patients and the general population [10]. Hence, we have investigated the perception of GPs toward acute stroke.

## Methods

### Study design and protocol

The study protocol was reviewed by the Regional Ethics Committee of the University of Debrecen (496-2018) and the Ethical Committee of the Health Research Council (51672/2018/EKU). Hungarian GPs and physicians enrolled in residency training programme for family medicine were invited to participate in the survey on a voluntary basis. The surveys, delivered on paper, were conducted by means of supervised self-administration during sessions for continuing medical education (GPs) or courses for the residency training programme (residents) between February 1, 2018, and July 31, 2018. The questionnaires were completed in an average of 19–28 min. Demographic variables (age, gender), characteristics of specialization (family medicine, residency, other board certification, years of practice), geographic area of practice (county, area code, type of settlement), and type of practice (adult or mixed practice attending adults and children) were recorded for each respondent.

We presented the participants with two clinical cases of acute stroke with risk factors, signs of middle cerebral artery stenosis, and duration of 60 min or for an unknown time period. To assess their perceptions regarding acute stroke management, two open-ended questions were presented. Question 1: what is your diagnosis for the case? Question 2: what would you tell your patient about their condition and what will happen to them?

Similar to the work of others [14], two clinical vignettes were developed by a board-certified neurologist with experience in acute stroke management. To assess if responders appropriately differentiated whether an acute stroke patient was eligible for intravenous thrombolysis or not, the time window for thrombolysis was the only factor varied. The clinical case scenarios describe textbook symptoms of acute stroke affecting the territory of the left middle cerebral artery. The indicated co-morbidities of the patients are the most prevalent risk factors for acute stroke [15].

#### Clinical vignette 1

Peter, a 54-year-old male patient has well-controlled hypertension, type 2 diabetes mellitus and atrial fibrillation. He arrived at the GP’s office accompanied by his wife at 8:30 A.M., with difficulty finding words, paresis of the right face (central facial palsy) and weakness of the right upper arm. His wife reported that her husband was well the previous night, the symptoms developed that morning at 7:30 A.M.

#### Clinical vignette 2

Peter, a 54-year-old male patient has well-controlled hypertension, type 2 diabetes mellitus and atrial fibrillation. He arrived at the GP’s office accompanied by his wife at 8:30 A.M., with difficulty finding words, paresis of the right face (central facial palsy) and weakness of the right upper arm. His wife reported that his speech was confusing the previous night prior to going to bed and the cup slipped out of his hand. He went to bed and they both noticed the symptoms as described since being awake this morning.

### Qualitative analysis

Responses were entered into Microsoft Excel spreadsheet and imported into the NVIVO 12 for Windows to assess word frequency of the given answers for each question and clinical case, separately [16, 17]. NVIVO was used to visualize qualitative data to support understanding. Formerly, others have also used narrative analysis in the stroke care setting as this method allows the assessment of language use, interpretation and reflexion regarding the content [18, 19]. Synonyms were collated and expletives and conjunctions were omitted. After data cleansing, word lists were translated to English and word clouds were compiled using NVIVO. Font sizes were proportional to word frequency.

### Quantitative analysis

For quantitative analysis, responses were categorised as correct or incorrect as indicated below.

Response to Question 1 Clinical Case 1 was considered correct in the following cases: stroke, stroke/embolism, stroke within time window, stroke (ischemic) in the territory of left middle cerebral artery, left-sided stroke, acute stroke, insufficient circulation in the area of middle cerebral artery (MCA), apoplexy, insufficient circulation in the territory of the left MCA, cerebral infarct, cerebral embolism in the left hemisphere, cerebrovascular event within 3 h, and cerebral embolism.

Response to Question 1 Clinical Case 2 was considered correct in the following cases: stroke, stroke outside of the time window (4.5 h), ischemic stroke outside the time window, left sided stroke, ischemic stroke in the area of MCA, stroke outside of the lysis time, insufficient circulation in the territory of cerebral artery, apoplexy (ischaemic), stroke in the territory of MCA, cerebral infarct/cerebral embolism (outside of time window), and subacute left lateral stroke.

Answers to Question 2 were assessed with respect to two dimensions, namely the presence or absence of urgency in communication and proper communication regarding eligibility for thrombolysis. Eligibility for thrombolysis was considered correct for the first case and incorrect for the second case, respectively. The answer was considered accurate if both dimensions were correctly included in the answers to Question 2.

To account for the potential impact that geographical location has regarding the diagnosis made by respondents, GP practices were characterised as being within or outside 90 km radius of the University of Debrecen. Debrecen was chosen as the centre for geographical reference as there has been a continuing joint effort to maintain an efficient acute stroke triage system since 2007.

Upon assessing the significant predictors for providing a correct diagnosis for Clinical Case 1, a priori variables (age and gender) and significant predictors identified by simple logistic regression were included in the initial model (diploma received before 2005, years of working, other specialisation, practice located within 90 km radius, inclusion of the possibility of intravenous thrombolysis in patient communication).

Upon assessing the significant predictors for providing a correct diagnosis for Clinical Case 2, a priori variables (age and gender) and significant predictors identified by simple logistic regression were entered in the initial model (board-certified GP, diploma received before 2005, years of working, other specialization, practice located within 90 km radius).

Significant predictors for providing accurate information (e.g. communicating urgency and relevant information about possible thrombolysis in response to Question 2) for Clinical Case 1 were included in a multiple logistic regression model; thus, a priori variables (age and gender) and significant predictors were identified by simple logistic regression and entered initially (board-certified GP, years of working, correct diagnosis (Question 1) for Clinical Case 1).

Significant predictors for providing accurate information (e.g. communicating urgency and relevant information about possible thrombolysis in response to Question 2) for Clinical Case 2 were included in a multiple logistic regression model; thus, a priori variables (age and gender) and significant predictors identified by simple logistic regression were entered initially (board-certified GP, diploma received before 2005, years of working, practice located within 90 km radius). Variables were entered in the model simultaneously and then factors not significantly contributing to the model were deleted. To assess the goodness of fit, a *χ*^2^ test for the final models was carried out.

Statistical analysis was performed with Stata 13.0 software (Stata Corporation). Values are given as mean ± SD or medians (with interquartile ranges), except for odds ratios (ORs), which are presented with their 95% confidence intervals.

## Results

### Respondents

Of the 127 respondents, 69 (54.3%) were women and the median age of the sample was 49 (34–62) years. The median years spent working after graduation was 14.5 (2–22.5) years; 98 (77.2%) physicians had board certification in family medicine and the remaining 29 (22.8%) were enrolled in the residency training programme for family medicine. Of the family medicine practitioners, 58 had additional board-certified specialisation (e.g. in internal medicine, neurology, anaesthesiology, etc.), while only 1 resident had prior board certification in internal medicine. Regarding the location of the practice with respect to its distance from Debrecen, 74 respondents (51 GPs and 23 residents) were located within the 90 km radius, while 44 (40 GPs and 4 residents) practiced outside this area (relevant geographic data was missing in 9 cases).

### Qualitative results

**Fig. 1:**
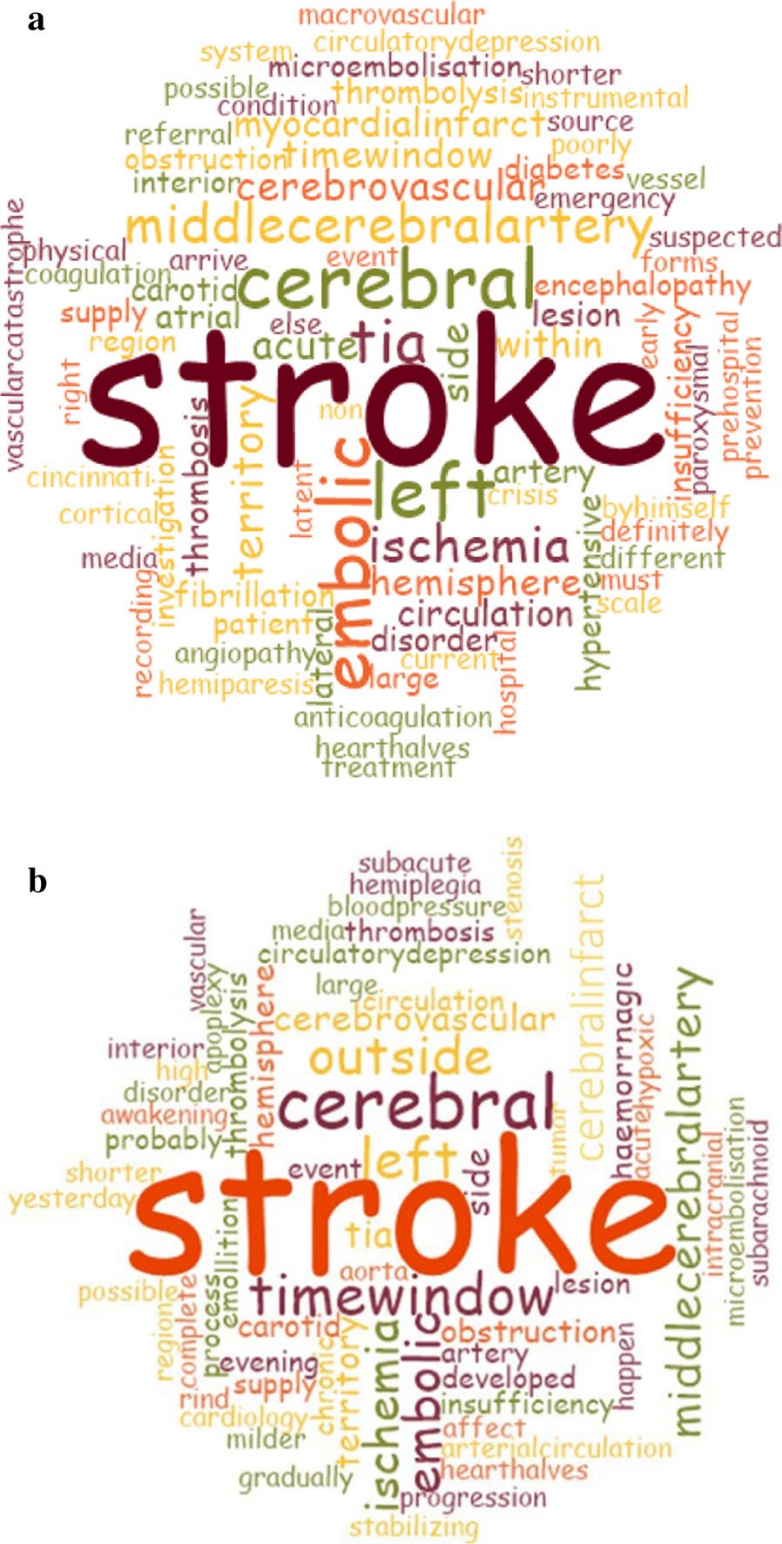
Word cloud of responses given to the question ‘What is your diagnosis for the case’ with respect to Clinical Case 1 (acute stroke patient *within time window, thrombolysis candidate*; **a**) and Clinical Case 2 (*outside the time window*; **b**). The top 100 most frequently used words included in the responses were used. The size of the words is proportional to their frequency (i.e. the most common word is the largest)

Qualitative analysis of responses to open-ended questions revealed stroke as the most frequent diagnosis for either case. Territorial localisation (middle cerebral artery, left) and possible aetiology (ischaemic, embolic) were indicated by many respondents as reflected by the word frequency for both clinical vignettes. Interestingly, the word ‘acute’ was only within the top 10 most frequent words for Clinical Case 1 describing a thrombolysis candidate, while the diagnosis for Clinical Case 2 was differentiated by frequent mentions of ‘outside’ and ‘time window’. It is of further interest that a transient ischaemic attack (TIA) was the fourth and tenth most frequently used word for Clinical Cases 1 and 2, respectively. Furthermore, it is also worthy of notice that cerebral infarct was also often indicated as a diagnosis for Clinical Case 2 (Fig. 1).

**Fig. 2:**
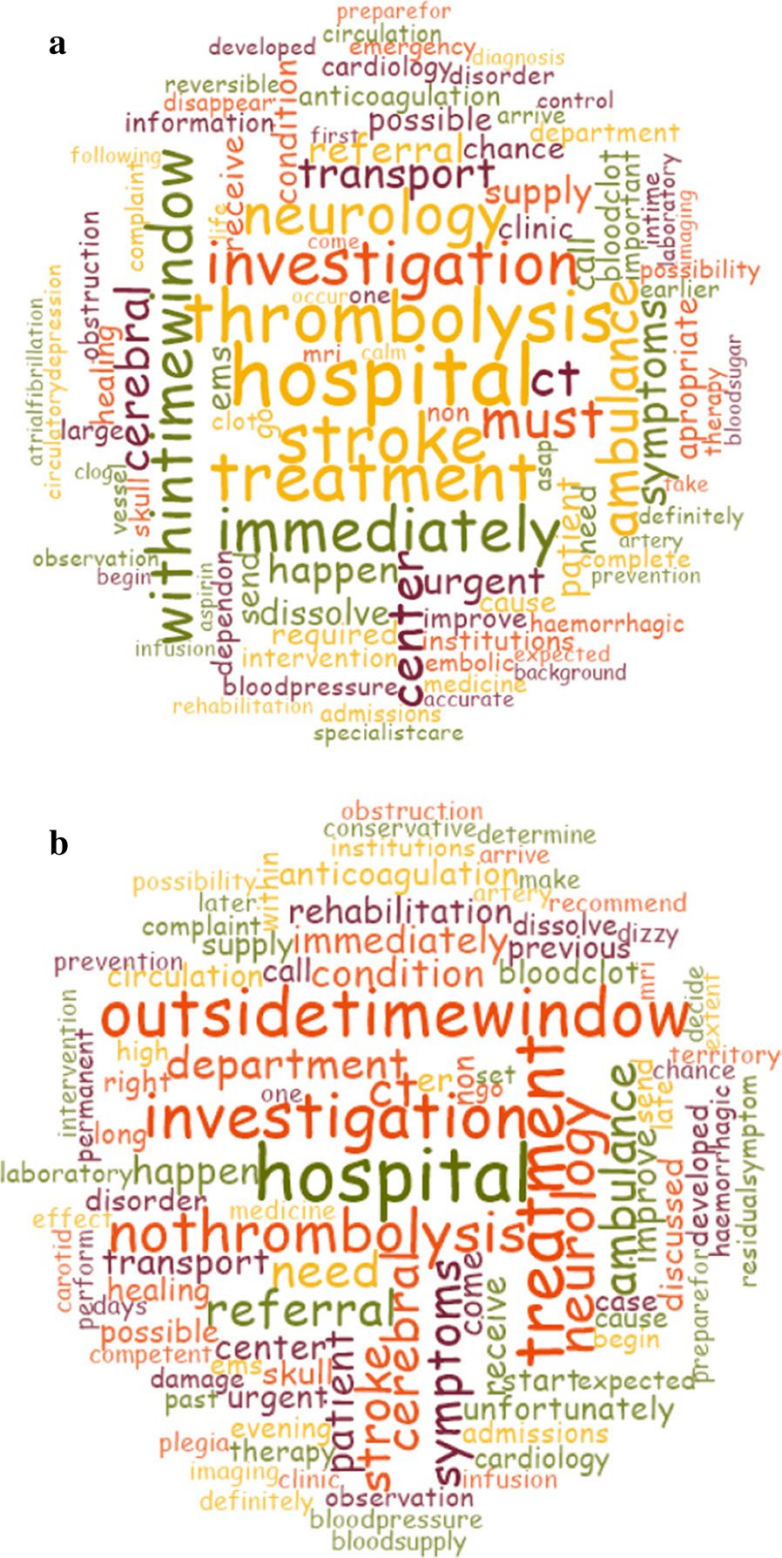
Word cloud of responses given to the question ‘What would you tell your patient about their condition and about what will happen to them?’ with respect to Clinical Case 1 (acute stroke patient *within time window, thrombolysis candidate*; **a**) and Clinical Case 2 (*outside the time window*; **b**). The top 100 most frequently used words included in the responses were used. The size of the words is proportional to their frequency (i.e. the most common word is the largest)

Regarding physician–patient communication, the respondents clearly distinguished between the two clinical scenarios in terms of eligibility for thrombolysis. The need for hospitalisation (most frequently used word in both cases) was articulated in both cases and the neurology department was frequently indicated in this context as well (eighth and sixth most frequent for Clinical Cases 1 and 2, respectively). Moreover thrombolysis (second), urgency (depicted by ‘within time window’ (sixth), immediately (seventh), must (ninth) and ambulance (tenth)) were articulated for Clinical Case 1, while ineligibility for thrombolysis (‘outside time window’ (third), ‘no thrombolysis’ (fifth)) was clearly communicated in Clinical Case 2. It should be further noted that the need (ninth) for referral (tenth), investigation (fourth) and CT scan (seventh) were also frequently mentioned with respect to patient communication in Clinical Case 2. Nonetheless, the word ‘stroke’ was not mentioned directly, rather it was circumscribed (cerebral infarct, insufficient cerebral circulation, shortage of blood flow, occlusion in the brain, blood clot clogging the arteries) (Fig. 2).

### Quantitative results

Simple logistic regression (used to identify factors significantly determining the odds for providing correct answers to Question 1 for Clinical Case 1) revealed that age is a significant predictor (OR 1.05, 1.02–1.09; *P* = 0.004). Additional significant predictors are indicated in Table 1. After adjusting for all significant predictors and a priori determinants by means of multiple logistic regression, we found that the odds for providing an inappropriate answer were significantly increased if the practice was outside the 90 km radius (OR 4.06, 1.35–12.24; *P* = 0.013) and if the physician omitted proper information about the possible eligibility for thrombolysis when communicating with the patient (OR 4.83, 1.55–15.07; *P* = 0.007). The additional significant predictors are indicated in Table 2. The final model proved to be significant by means of goodness-of-fit *χ*^2^ statistic (*P* = 0.48).

**Table 1:** Simple logistic regression of factors significantly determining the odds for providing a correct answer to Question 1 for Clinical Cases 1 and 2

Diagnosis	Odds ratio	95% Confidence interval		*P*
		Lower limit	Upper limit	
Clinical Case 1				
Gender (Woman/Man)	1.213	0.525	2.802	0.651
Age (years)	1.050	1.016	1.086	0.004
Diploma received before 2005 (yes/no)	0.321	0.112	0.916	0.034
Years of working (years)	1.058	1.019	1.100	0.003
Other specialisation (yes/no)	0.292	0.120	0.710	0.007
Practice located within 90 km radius (yes/no)	4.504	1.779	11.401	0.001
Thrombolysis mentioned to patient (yes/no) (Q2 case 1)	3.976	1.702	9.289	0.001
Thrombolysis communicated accurately (yes/no) (Q2 case 1)	2.833	1.165	6.892	0.022
Clinical Case 2				
Gender (Woman/Man)	1.133	0.484	2.656	0.773
Age (years)	1.055	1.019	1.092	0.002
Work (board-certified family physician) (yes/no)	0.085	0.011	0.654	0.018
Diploma received before 2005 (yes/no)	0.104	0.023	0.467	0.003
Years of working (years)	1.077	1.034	1.122	0.000
Other specialisation (yes/no)	0.391	0.163	0.940	0.036
Practice located within 90 km radius (yes/no)	3.631	1.454	9.065	0.006

**Table 2:** Significant predictors and a priori determinants for providing correct diagnosis with respect to Clinical Case 1, final multiple logistic regression model

Diagnosis 1	Odds ratio	95% Confidence interval		*P*
		Lower limit	Upper limit	
Gender (Woman/Man)	0.540	0.176	1.662	0.283
Age (years)	0.996	0.930	1.067	0.911
Years of working (years)	1.033	0.955	1.112	0.417
Practice located within 90 km radius (yes/no)	4.064	1.350	12.237	0.013
Thrombolysis mentioned correctly –Question 1	4.834	1.550	15.075	0.007

Simple logistic regression (used to identify factors significantly determining the odds for providing correct answers to Question 1 for Clinical Case 2) showed that age is a significant predictor (OR 1.05, 1.02–1.09; *P* = 0.002). Additional significant predictors are indicated in Table 1. The final multiple logistic regression model showed that the odds for providing an inappropriate answer were three times greater if the practice was outside the 90 km radius (OR 3.03, 1.06–8.61; *P* = 0.038). The final model was significant (*P* = 0.22) (Table 3).

**Table 3:** Significant predictors and a priori determinants for providing correct diagnosis with respect to Clinical Case 2, final multiple logistic regression model

Diagnosis 2	Odds ratio	95% Confidence interval		*P*
		Lower limit	Upper limit	
Gender (Woman/Man)	0.985	0.357	2.718	0.977
Age (years)	0.995	0.927	1.069	0.900
Years of working (years)	1.074	0.993	1.162	0.072
Practice located within 90 km radius (yes/no)	3.027	1.064	8.610	0.038

When analysing the factors that contribute to the accuracy of physician–patient communication in Clinical Case 1, both age and gender proved to be significant upon simple regression analysis (Table 4). Interestingly, the final model contained only these a priori-identified variables (age: OR 1.05, 1.02–1.08; *P* = 0.01, and gender: OR 2.73, 1.23–6.06; *P* = 0.013); the model was significant (*P* = 0.41).

**Table 4:** Simple logistic regression of factors significantly determining the odds for providing accurate answer to Question 2 for Clinical Cases 1 and 2

Accuracy	Odds ratio	95% Confidence interval		*P*
		Lower limit	Upper limit	
Clinical Case 1				
Gender (Woman/Man)	3.284	1.541	6.999	0.002
Age (years)	1.051	1.023	1.080	0.000
Work (board certified family physician) (yes/no)	0.206	0.762	0.559	0.002
Years of working (years)	1.062	1.026	1.100	0.001
Diagnosis	2.833	1.165	6.892	0.022
Clinical Case 2				
Gender (Woman/Man)	1.360	0.486	3.802	0.558
Age (years)	1.082	1.036	1.130	0.000
Work (board-certified family physician) (yes/no)	0.189	0.067	0.535	0.002
Diploma received before 2005 (yes/no)	0.222	0.790	0.623	0.004
Years of working (years)	1.087	1.024	1.153	0.006
Practice located within 90 km radius (yes/no)	3.795	1.031	13.967	0.045

Significant predictors yielded by simple logistic regression for Clinical Case 2 (describing an acute stroke patient who is out of the ‘time window’) regarding accurate physician–patient communication are shown in Table 4. The final multiple model contained only one significant factor, that is, age (OR 1.15, 1.02–1.29; *P* = 0.019); the final model was significant (*P* = 0.094).

## Discussion

Our findings suggest that the translation process for intravenous thrombolysis candidacy in acute stroke management was successful in the specific geographical region of Hungary investigated. These conclusions are drawn based on how GPs assessed the two acute stroke clinical scenarios presented (Figs. 1 and 2).

The vast majority of respondents clearly identified the two cases as stroke, considered thrombolysis and differentiated between the two scenarios in this respect (eligibility or lack of eligibility for thrombolysis) (Figs. 1 and 2). Furthermore, it seems that the concept of acute stroke as an emergency also became acknowledged as time progressed. Implicitly our results also indicated that GPs/residents take notice of atrial fibrillation as a possible risk factor for stroke as an embolic origin was often indicated. Our results show a more favourable scenario regarding the translation of acute stroke guidelines than that described by prior investigators. In a similar survey from Brazil assessing the perception of 149 EMS professionals towards stroke, the authors found that a great majority of responders were able to properly diagnose the presented case as stroke; furthermore, ‘time window’ was only mentioned once, the ‘possibility for thrombolysis’ was mentioned four times and the ‘need for CT scans’ twelve times [14]. Conversely, more recent results have also shown insufficient knowledge of community GPs from China with respect to pre-hospital stroke care, a finding paralleled by low intravenous thrombolysis rates; thus, the need for mastering pre-hospital stroke recognition to reduce prehospital delays was emphasized by the authors [20]. The relevance of these findings are profound as both countries organise acute stroke care in agreement with the international guidelines [14, 20]. A recent Hungarian prospective study presented 250 consecutive stroke patients ineligible for thrombolysis admitted to a hospital devoid of a dedicated stroke unit. The findings have shown that 37.2% of these patients first contacted the GP on duty or went to visit the GP’s office (47 and 46 patients, respectively, totaling 93 patients) and only 91 (36.4%) patients called the EMS immediately [21]. Moreover, the authors found a 2.66 increase of odds for contacting the EMS if the patient was known to have atrial fibrillation, offering the speculative notion that patients managed with stroke risk disorders are more aware of the most favourable course of action in acute stroke as physician–patient encounters may offer appropriate grounds for patient education.

Our survey has shed light on the unresolved issues regarding the definition and consequent uncertainties related to the needed diagnostic work-up of TIA. Previously, an operational definition was used for TIA, diagnosed as any focal cerebral ischaemic event lasting less than 24 h. This definition was however refined to focus on the lack of acute infarction additional to the focal cerebral ischemic event [22]. The need for redefining TIA was called for in light of accumulating evidence that the traditional definition may impede timely initiation of acute stroke therapy. Furthermore, the frequency distribution of TIA also underlined the need to replace the arbitrarily defined 24-h long cut off used in the definition as 60% and 71% of MRI-verified TIA patients recovered within 1 and 2 h, respectively, while only 14% recovered 6 h after the onset of symptoms [22]. Accordingly, TIA was redefined and, in light of the new definition, a diagnosis of TIA is not possible in the absence of neuroimaging procedures. Hence, the frequent diagnosis of TIA in the case presented in these two clinical vignettes reflects a point for further intervention. This is further accounted for if one acknowledges that the tissue-based definition of TIA was only recently incorporated into the Hungarian practice guidelines for the management of acute ischaemic stroke in September 2017 [23]. The difficulty to diagnose and manage TIAs by primary care physicians is well established [24]. A Swiss survey assessed the referral rate of GPs for TIA after a targeted campaign that increased awareness regarding TIA management and found that although the estimated risk for stroke after TIA within 24 h and 3 months was generally overestimated, only about half of the responding GPs would refer their patient immediately for rigorous work-up [25].

Quantitative assessment of the data showed that having the practice located within 90 km of Debrecen increases the likelihood of proper diagnosis in both clinical scenarios. The significance of this geographical distinction comes from the vigorous acute stroke triage system developed in this region driven by the secondary stroke centre of the Department of Neurology, University of Debrecen. Additional to well-circumscribed patient-referral pathways supported by formalised agreement between relevant actors of regional acute stroke care (EMS, other regional hospitals, GPs), systematic monitoring of clinical decisions for thrombolysis eligibility and outcomes were supplemented by continuing education for GPs, paramedics, oxyologists and the in-hospital stroke team. The success of the programme is reflected by outstanding rates for intravenous thrombolysis, reaching 16–19% of acute stroke cases [26], and the lower stroke-related mortality in Hajdu-Bihar county (7.07 per 100,000 inhabitants versus the country average of 8.2 per 100,000 inhabitants in the county of Debrecen) in 2018 [27].

When assessing the content of patient–physician communication regarding the inclusion of urgency and relevant information for thrombolysis, age was a significant predictor for both clinical vignettes. An increase in age by 1 year contributed to a 1.04- and 1.15-fold increase of odds for incorrect responses in Clinical Cases 1 and 2, respectively. This age-related effect may be accounted for by the fact that medical knowledge and clinical practice evolves over time. Prior work has shown a less positive attitude toward use of guidelines among older GPs [28], an effect that may contribute to insufficient translation of policy to practice.

Furthermore, male physicians were more likely to answer incorrectly in the event that the patient described was not a candidate for thrombolysis (Clinical Case 2). This finding is in contradiction with a previous study, where adherence to practice guidelines was estimated with the inclusion of primary care physicians who rated video vignettes portraying coronary heart disease. The authors were unable to identify a main or interaction effect regarding compliance with guidelines in terms of gender for the physician [29].

Previous work shed light on the significant effect that perceptions for prognosis have on limiting recommendations for managing the acute intracerebral stroke patient. In a sample of 742 neurosurgeons and neurologists, the odds for limiting treatment was increased by 1.61 (95% confidence interval 1.12–2.33) when a prognosis of 0% for independent living was predicted by a prognostic scale. Conversely, the likelihood for treatment limitations was lower if the prognostic score estimated a high chance of independence [30]. Corroborating findings regarding the association between stroke knowledge and outcome expectations with respect to the importance of rapid stroke identification were reported in a previous study. Here, greater stroke knowledge increased the likelihood for attributing more importance to the rapid identification of stroke symptoms (OR, 1.23; 1.002–1.51) [31].

This study has some limitations. Respondents were approached at an event for continuing medical education or seminar organised within the frame of residency training; hence, participants were motivated to learn. Nonetheless, these events were not dedicated to acute stroke care and hence prior knowledge was assessed. Moreover, using open-ended questions to assess the approach to the clinical case enabled the assessment of clinical competencies as it mandates higher order thinking and knowledge construction [32]. Limited sample size must also be acknowledged; however, robust results were yielded regardless.

## Conclusion

Our main findings show that policy changes regarding the appropriate determination of candidacy for intravenous thrombolysis in the acute stroke care setting are acknowledged by GPs; hence, this group of physicians are a good target for raising awareness to the acute stroke-related issues. Physician level attributes, including the physician’s age, gender, need for continuous medical education and professional development, were also identified. Furthermore, we found a clear geographical effect reflective of a regional, formalised acute stroke triage system, calling attention to the beneficial effect of a systematic approach to translational efforts.

## Data Availability

The datasets used and/or analysed during the current study are available from the corresponding author on reasonable request.

## Abbreviations

EMS: Emergency medical service
GPs: General Practitioners
MCA: Middle cerebral artery
TIA: Transient ischemic attack
OR: Odds ratio
